# Standardized mirror confrontation: Body-related emotions, cognitions and level of dissociation in patients with Posttraumatic Stress Disorder after childhood sexual abuse

**DOI:** 10.1186/2051-6673-1-10

**Published:** 2014-07-25

**Authors:** Elisabeth Borgmann, Nikolaus Kleindienst, Silja Vocks, Anne Sibilla Dyer

**Affiliations:** Department of Psychosomatic Medicine and Psychotherapy, Central Institute for Mental Health, Heidelberg University, J5, D-68159 Mannheim, Heidelberg, Germany; Clinical and Biological Psychology, Faculty for Social Sciences, Mannheim University, D-68131 Mannheim, Germany; Clinical Psychology and Psychotherapy, Osnabrück University, Knollstrasse 15, D-49069 Osnabrück, Germany

**Keywords:** Body image, Posttraumatic Stress Disorder, Childhood sexual abuse, Mirror confrontation, Emotions, Dissociation

## Abstract

**Background:**

A criterion for Posttraumatic Stress Disorder (PTSD) is the avoidance of trauma-associated stimuli that trigger emotional suffering. First studies on body image of patients with PTSD after childhood sexual abuse (CSA) support the hypothesis that awareness of the own body triggers emotional suffering.

**Methods:**

Body-related emotions, cognitions and level of dissociation of n = 17 patients meeting DSM-IV criteria for PTSD and n = 29 healthy controls (HCs) during a standardized mirror confrontation while wearing a standard bikini were assessed.

**Results:**

It was shown that expecting to be and while being confronted with one’s own body, patients with PTSD showed significantly stronger negative emotionality and cognitions as well as higher dissociative states as compared to HCs.

**Conclusions:**

Findings suggest that in patients with PTSD after CSA, one’s own body might function as a stimulus that leads to aversive emotional responses, negative cognitions and dissociative states. The elaboration of treatment for PTSD should consider these body-related aspects, e.g., by investigating the effects of body exposure.

## Background

The fifth revision of the Diagnostic and Statistical Manual of Mental Disorders (DSM-5) classifies PTSD no longer as an anxiety disorder, but as a trauma and stressor-related disorder [1]. Several scholars had criticized the former understanding of PTSD as an anxiety disorder with anxiety as the core emotion e.g. [2] as too narrow and postulated a revision. Budden [3] hypothesizes that shame plays a central role in PTSD because it underlies peri-traumatic and traumatic experiences. Stone [4] and Budden [3] speak in general of PTSD as a “disturbance of affect systems”. According to Glover [5] and Reynolds and Brewin [6] shame, guilt, anger, sadness, and mistrust are the most widely reported emotions in PTSD. Intense levels of emotions such as shame and anger were reported to be associated with the trauma [7].

In a study by Finucane [8], the extent of five emotions, assessed by the trait version of the Basic Emotion Scale, was compared across four samples, i.e., patients with PTSD, with chronic pain and depression as well as HCs. Only the PTSD group experienced all negative emotions, i.e., fear, anger, sadness and disgust, more frequently than did HCs. Additionally, disgust was reported in patients with PTSD more often than in the other groups. Following fear, anger was the emotion that patients with PTSD reported most frequently [8].

CSA significantly increases the probability to develop PTSD [9–13]. Avoidance of trauma-associated stimuli that trigger intrusions and emotional suffering is a criterion for PTSD. Especially after the experience of CSA, awareness of the own body or body-related sensations might function as cues triggering intrusions and emotional suffering. Studies on body image in PTSD support this hypothesis e.g. [14, 15]. Dyer et al. [16] found that the cognitive-affective and behavioral components of body image of female participants with PTSD after CSA are impaired, independently of a comorbid eating disorder. The quoted studies have addressed body related attitudes and behaviors, however these were only assessed in retrospect. So far, no study investigated body-related emotional and cognitive reactions in a controlled situation under standardized conditions.

The aim of the present study was to assess the extent and course of body-related emotions and thoughts as well as the level of acute dissociation in patients meeting criteria for PTSD after CSA during exposure to a mirror in comparison to HCs.

It was assumed that patients with PTSD show stronger negative emotions and cognitions related to one’s own body and suffer more from acute dissociative symptoms than HCs when being confronted with their mirrored body. Finally, the course of specific emotions during the mirror confrontation in patients with PTSD was explored.

## Methods

### Participants

Within the in- and outpatient units at the Department of Psychosomatic Medicine and Psychotherapy, Central Institute of Mental Health, Mannheim, Heidelberg University (Germany), 28 patients fulfilled inclusion criteria for the present study. Of those, 11 refused participation. Altogether, 17 patients with PTSD after CSA were recruited. HCs were enlisted by press advertisement (n = 29).

Inclusion criteria for the clinical group were the diagnosis of PTSD as assessed by the Structured Clinical Interview for DSM-IV Axis I (SCID-I) [17] and a value above the cut-off of 5 on the subscale “Sexual abuse” of the Childhood Trauma Questionnaire (CTQ) [18, 19], which indicates the experience of sexual abuse in childhood. Further inclusion criteria were female sex, an age between 18 and 65 years and good knowledge of the German language. In order to be included in the group of HCs, criteria of any mental diagnosis (as assessed by SCID-I) and of CSA (value below 5 on the subscale sexual abuse of the CTQ) must not be fulfilled. Exclusion criteria for the two groups were acute substance abuse or substance dependence, borderline personality disorder, acute psychosis, bipolar-I disorder, acute severe major depression, neurologic or sensory disorders as well as organic disorders, which might influence regular brain function.

Within the PTSD group, 5 patients were diagnosed with co-occurring bulimia nervosa. No other lifetime or current eating disorder was diagnosed. All PTSD participants were currently in psychiatric treatment. Previously, 14 PTSD participants reported to have been in diverse psychiatric treatments. According to the participants self-report, the sexual abuse started on average at the age of 9.20 (SD = 5.09). The time lag between the most stressful traumatic event and the examination was 23.87 years (SD = 12.19).

The institutional review boards of the University of Heidelberg-medical faculty approved the study, and written informed consent from each participant was obtained. Patients and controls received €20 payment for participation in the study.

### Standardized mirror confrontation

The procedure of mirror exposure was strictly standardized and followed an adapted version of the protocol originally developed for eating disorders [20, 21]. According to the protocol, the investigator instructed the participant to look at twelve areas (hair, face, shoulder, upper arms, forearms, hands, breasts, waist, hip, thighs, legs, feet) of one’s own body in a fixed sequence (see Appendix 1), focusing each area for 50 seconds.

### Measures

The scale “Sexual abuse” from the *Childhood Trauma Questionnaire* (CTQ) [18, 19, 22] was applied in the present study which measures the severity of sexual abuse in childhood. In the present study, the internal consistency for the subscale was excellent (Cronbach’s α: .974).

Furthermore, the subscale “symptom severity” from the *Posttraumatic Diagnostic Scale* (PDS) [23, 24] was applied for the assessment of the PTSD-symptomatology. The internal consistency of the subscale was excellent in the present study (Cronbachs α = .910).

In order to assess negative body-related emotions over the course of the standardized mirror confrontation, the *Emotions Rating* (ER, modified after Vocks et al. [20]) was applied. The ER assesses the extent of sadness, tension, anxiety, disgust, anger, shame, guilt, and aversive tension on a Likert-scale ranging from 0 to 10. A composite score of negative body-related emotionality was calculated by taking the mean value over the assessed emotions for each assessment point. The internal consistency was excellent in the present study (Cronbach’s α = .991).

The repeated assessment of extent of dissociation during the standardized mirror confrontation was measured by the *Dissociation-Distress Scale* (DSS-4) [25] which is a short version of the DSS-21 [26]. It assesses the extent of pathologic dissociation and distress. In the present study, the internal consistency was good (Cronbach’s α = .899).

Finally, the *Thoughts Checklist* (TC) [27] was applied after the body exposure session. The TC consists of 17 items measuring negative cognitions regarding one’s own body. The internal consistency was excellent (Cronbachs α = .926).

### Procedure

Right after the written informed consent had been obtained, baseline assessment was accomplished which included the ER, the CTQ-sexual abuse subscale and the PDS-symptom severity subscale. The participants were accompanied by a female investigator into the lab. Confrontation-pre assessments (DSS-4; ER) were conducted. Subsequently, the participant was left alone to change her clothes against a standardized pink bikini which was available in several sizes and a bath robe. The investigator came back into the lab and sat down with her back turned to the participant who was asked to stand in front of a full-length mirror. Then, the DSS-4 and the ER were administered to the participants again (confrontation-start). Afterwards, the body confrontation was started. Immediately after the mirror confrontation, while still standing in front of the mirror, the participant was again asked to answer the questions of the DSS-4 and the ER (confrontation-end). After the investigator left the room, the participant got dressed. The investigator came back into the lab and conducted the post-assessment including the ER, the DSS-4 and the TC.

### Data Analysis

Differences between the groups regarding age, BMI, sexual abuse (as measured by the CTQ-sexual abuse subscale) and severity of symptomatology (as measured by the PDS-subscale) were tested through a one-factorial ANOVA. Mann–Whitney-U-Tests were used to analyze differences in the ER, the DSS-4 and the TC between the groups. Spearman´s rho (r_S_) was used for correlation calculation. For both groups Friedman tests were used to test for differences in the level of negative body-related emotionality and for the level of specific body-related emotions. Pairwise comparisons between different assessment points were carried out with Wilcoxon Tests for ordinal data and with McNemar tests for binary data.

## Results and discussion

No significant difference was found between the two groups regarding age and BMI (Table 1). In line with our definitions of groups, significant differences between groups were shown for the CTQ-sexual abuse subscale and the PDS-symptom severity subscale. The scores on the ER, DSS-4 and the TC were not normally distributed.

**Table 1:** **Mean and standard deviation of age, BMI, CTQ and PDS for healthy controls (HCs) and patients with Posttraumatic Stress Disorder (PTSD)**

		Age	BMI	CTQ-subscale sexual abuse	PDS-subscale symptom severity
HCs	M	32.83	26.125	0.138	3.357
(n = 29)	SD	12.204	5.851	0.441	4.088
PTSD	M	37.59	28.726	15.000	33.200
(n = 17)	SD	6.865	8.255	5.679	11.175
F		3.277	1.799	199.751	82.749
df		1	1	1	1
p		.148	.234	< .001	< .001

### Group differences in emotions and dissociation

Even after Bonferroni-adjustments, Mann–Whitney-U-Tests showed that, compared to HCs, patients with PTSD had significantly higher scores on the ER and DSS-4 at all assessment points (Table 2 and 3). In post-hoc tests the potentially confounding effects of age, BMI and eating disorders were partialized out. The post-hoc tests revealed statistically significant differences between groups regarding negative body-related emotionality and dissociation for all assessment points, except for the DSS-4 at confrontation-pre. At confrontation-end, the ER score was significantly positively correlated with PTSD severity (r_S_ = .546, p = .035), but not with severity of childhood sexual abuse within the PTSD group. The DSS-4 score is positively associated with PTSD severity to confrontation-start (r_S_ = .724, p = .002) as well as confrontation-end (r_S_ = .630, p = .012).

**Table 2:** **Comparison of means and standard deviations of the composite score of negative body-related emotionality across all assessment points**

		Baseline	Confrontation pre	Confrontation start	Confrontation end	Post assessment
HCs	M	1.901	0.434	0.616	0.581	0.241
(n = 29)	SD	2.705	0.314	0.683	0.765	0.411
PTSD	M	4.524	4.345	6.084	6.597	5.387
(n = 17)	SD	2.310	2.273	2.691	2.867	3.108
Z		-3.036	-5.023	-4.953	-5.049	-5.235
p		.001	< .001	< .001	< .001	< .001

**Table 3:** **Comparison of means and standard deviations of dissociation across assessment points**

		Confrontation pre	Confrontation start	Confrontation end	Post assessment
HCs	M	0.069	0.052	0.035	0.000
(n = 29)	SD	0.371	0.279	0.088	0.000
PTSD	M	1.485	2.382	3.456	2.338
(n = 17)	SD	1.997	2.064	3.195	2.537
Z		-4.153	-4.857	-4.393	-5.128
p		< .001	< .001	< .001	< .001

### Group differences in body-related cognitions

Significant differences were detected between patients with PTSD and HCs regarding the TC which is an indicator for negative body-related thoughts during the standardized mirror confrontation. The thoughts of patients with PTSD (M = 63.941; SD = 18.806) were significantly (Z = -4.627; p < .001) more negative than those of HCs (M = 29.393; SD = 9.410).

These results were fully corroborated from analyses accounting for the potentially confounding effect of age, BMI and co-occurring eating disorder.

### Course of negative emotionality and specific emotions in patients PTSD after CSA

**Figure 1:**
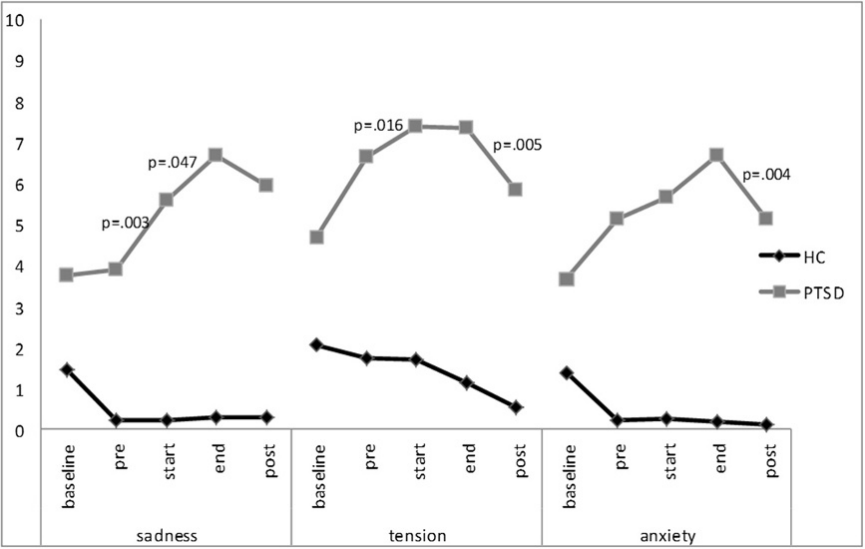
**Course of the emotions (sadness, tension, anxiety) across assessment points in patients with Posttraumatic Stress Disorder (PTSD) after childhood sexual abuse compared to healthy controls (HCs).**

**Figure 2:**
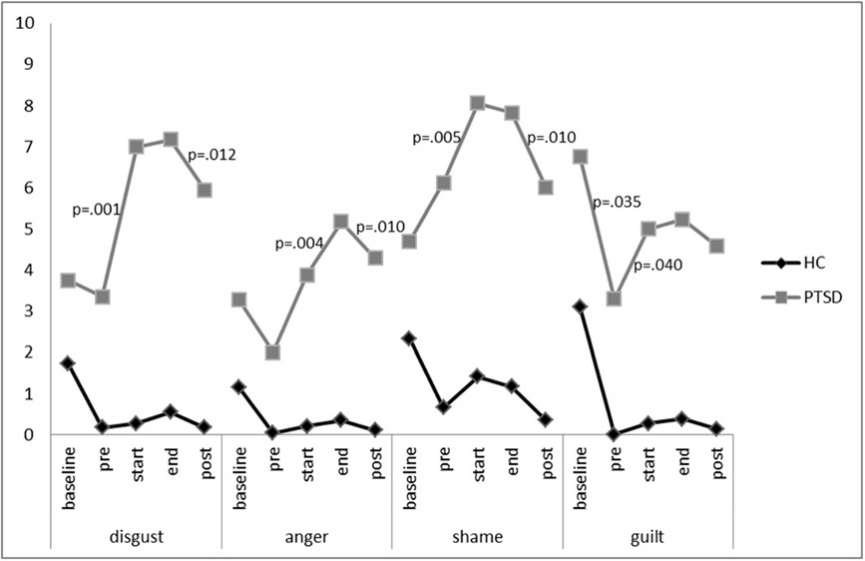
**Course of the emotions (disgust, anger, shame, guilt) across assessment points in patients with Posttraumatic Stress Disorder (PTSD) after childhood sexual abuse compared to healthy controls (HCs).**

Differences in the levels of the ER composite score in patients with PTSD exist between assessment points (χ^2^ (4) = 26.277; p < .001). Wilcoxon tests show no significant difference in negative emotionality of PTSD participants between baseline and confrontation-pre (Z = -.402; p = .687). Negative emotionality in PTSD participants rises significantly between confrontation-pre and confrontation-start (Z = -3.52; p < .001) as well as between confrontation-start and confrontation-end (Z = -2.420, p = .016). Between confrontation-end and post-assessment, negative emotionality of PTSD participants is reduced significantly (Z = -3.243, p = .001). For every specific emotion, Friedman tests revealed significant differences between assessment points for participants with PTSD. Figure 1 and 2 show the course of specific emotions over assessment points. Participants with PTSD after CSA reported a significant decrease of guilt from baseline to confrontation-pre (Z = -2.109; p = .035). For none of the assessed emotions, a significant increase was shown for this interval. Over the course of assessment points, all patients with PTSD showed an increase in the extent of every single emotion from confrontation-pre to confrontation-end. From confrontation-pre to confrontation-start, patients with PTSD experienced a significant increase in sadness (Z = -2.955; p = .003), tension (Z = -2.412; p = .016), disgust (Z = -3.306; p = .001), shame (Z = -2.820; p = .005) and guilt (Z = -2.057; p = .040), but not for anxiety (Z = -.630; p = .528). A significant increase was shown from confrontation-start to confrontation-end for sadness (Z = -1.990; p = .047) and anger (Z = -2.844; p = .004). The extent of tension, disgust, shame and guilt did not change significantly between these two assessment points. All patients with PTSD showed a decrease in the extent of all assessed emotions from confrontation-end to post assessment. The extent of tension (Z = -2.831; p = .005), anxiety (Z = -2.857; p = .004), disgust (Z = -2.511; p = .012), anger (Z = -2.565; p = .01) and shame (Z = -2.569; p = .010) was significantly reduced from confrontation-end to post assessment. The extent of sadness and guilt did not differ significantly between confrontation-end and post assessment. At post-assessment, the extent of sadness (Z = -3.205; p = .001), disgust (Z = -3.087; p = .002) and anger (Z = -2.301; p = .021) were significantly higher compared to confrontation-pre.

### Course of negative emotionality and specific emotions in healthy controls

Regarding the ER composite score in HCs, significant differences between assessment points were found (χ^2^ (4) = 17.428; *p* = .002). Wilcoxon tests reveal a significant decrease in negative emotionality for HCs between confrontation-end and post assessment (Z = -3.437; p = .001). The following emotions decreased significantly in HCs between confrontation-end and post assessment: tension (Z = -2.913; p = .004), disgust (Z = -2.428; p = .015); anger (Z = -2.070, p = .038) and shame (Z = -3.384; p = .001).

### Differences in Course of Emotions between groups

From baseline to confrontation-pre, no significant difference between patients with PTSD and HCs regarding an increase of the composite score of the ER was shown (p = .210). From confrontation-pre to confrontation-start, the composite score of the ER raised significantly (p = .003) more often in patients with PTSD than in HCs. Furthermore, in patients with PTSD, a decrease in the composite score of the ER from confrontation-end to post assessment appeared significantly more often than in HCs (p = .007). The most rapid shift of ER was found between confrontation-pre to confrontation-start. The rate of change is significantly higher in the PTSD group in comparison to the HC group (p < .001).

The results of the present study suggest that, in contrast to HCs, patients with PTSD after CSA display significantly stronger negative emotional and cognitive reactions as well as higher dissociative states in response to viewing one’s own body in the mirror. Although negative emotions were already significantly higher in patients with PTSD compared to HCs at baseline and at confrontation-pre, they further increased significantly more often in patients with PTSD from confrontation-pre to the time when participants catch their own reflection for the first time (confrontation-start). For patients with PTSD, the course of an increase in emotional extent from confrontation-pre to –start and a decrease from confrontation-end to post assessment was shown for all assessed emotions; for tension, disgust and shame, the differences between these assessment points were significant. These results point to the relevance of a broad range of body-related emotions in PTSD.

Our results implicate an exaggerated emotional responsivity to looking at one’s own body in PTSD. However, since we did not use a non-body image-related control task, the specificity of this result is limited, i.e., it is not clear whether the enhanced reactivity is a specific response to the mirror confrontation or whether it reflects a general emotional hyperresponsivity in the patients. Previous research provided hints for such a general emotional hyperresponsivity, as Finucane et al. [8] demonstrated a higher frequency of emotions occurring under daily life conditions in patients with PTSD. However, results of the present study extend the findings by Finucane et al. [8] insofar as they demonstrate an enhanced emotional reaction to a specific standardized stimulus, i.e., one’s own body. One’s own body might be of specific importance in the patient group examined in the present study, as all participants were survivors of CSA. Nevertheless, with the design of the present study, it cannot be determined whether and to what degree the emotional reaction to mirror exposure exceeds the reaction to non-disorder specific triggers.

In addition, our results of a stronger experience of disgust and shame in patients with PTSD when being contrasted to HCs are in line with previous research also indicating the prominent role of the emotions of disgust [28, 8] and shame [3] in PTSD. These former studies had shown strong feelings of disgust and shame in patients with PTSD on a trait-basis, our results extend these findings in the sense, that patients with PTSD after CSA showed stronger feelings of body-related disgust and shame compared to HCs in the specific situation of mirror confrontation.

When interpreting the results of the present study, it has to be considered that the generalizability of the results might be limited by the small number of participants as well as the focus on female survivors of CSA with PTSD. Male patients with PTSD as well as healthy survivors of CSA or patients with PTSD related to other kinds of interpersonal violence during childhood were not included in the study. However, the results might be meaningful for clinical practice.

## Conclusions

Our results suggest that in patients with PTSD after CSA, one’s own body might function as a stimulus that leads to aversive emotional responses, negative cognitions and dissociative states. Despite of the increasing evidence of body-related emotional and cognitive problems in patients with PTSD after CSA, manual driven cognitive behavioral therapies e.g. [29–31] do not incorporate body-related interventions so far. Therefore, it might be promising to include elucidation about body-related aversive emotions in a PTSD-CSA educational program. Normalizing these negative body-related emotional reactions by educating the patients that most of female CSA survivors display similar emotional and cognitive experiences might at least reduce secondary shame. Furthermore, focusing body-related emotions during cognitive restructuring or mirror exposure procedures as it is part of cognitive-behavioral body image treatments (e.g., for eating disorders: Vocks et al. [21] might be an important adjunct to standard trauma-focussed therapies.

## Abbreviations

BMI: Body Mass Index
CSA: Childhood Sexual Abuse
CTQ: Childhood Trauma Questionnaire
DSS-4: Dissociation Distress Scale-4
ER: Emotions Rating
HCs: Healthy Controls
PDS: Posttraumatic Diagnostic Scale
PTSD: Posttraumatic Stress Disorder
TC: Thought Checklist.

## References

[1] American Psychiatric Association (2013). Diagnostic and statistical manual of mental disorders.

[2] Kunst MJJ, Winkel FW, Bogaerts S (2011). Posttraumatic anger, recalled peritraumatic emotions, and PTSD in victims of violent crime. J Interpers Violence.

[3] Budden A (2009). The role of shame in posttraumatic stress disorder: A proposal for a socio-emotional model for DSM-V. Soc Sci Med.

[4] Stone AM (1992). The role of shame in post-traumatic stress disorder. Am J Orthopsychiat.

[5] Glover H (1988). Four syndromes of post-traumatic stress disorder: stressors and conflicts of the traumatized with special focus on the Vietnam combat veteran. J Traum Stress.

[6] Reynolds M, Brewin CR (1999). Intrusive memories in depression and posttraumatic stress disorder. Behav Res Ther.

[7] Brewin CR, Andrews B, Rose S (2000). The Relation of Fear, Helplessness and Horror to Posttraumatic Stress Disorder: Investigating DSM-IV Criterion 2. J Traum Stress.

[8] Finucane AM, Dima A, Ferreira N, Halvorsen M (2012). Basic emotion profiles in healthy, chronic pain, depressed and PTSD individuals. Clin Psychol Psychother.

[9] Breslau N, Kessler RC, Chilcoat HD (1996). Trauma and posttraumatic stress disorder in the community: the, Detroit Area Survey of Trauma. Arch Gen Psychiatry.

[10] Kessler R, Sonnega A, Bromet E (1995). Posttraumatic stress disorder in the national comorbidity survey. Arch Gen Psychiat.

[11] Kessler RC, Zhao S, Katz SJ, Kouzis AC, Frank RG, Edlund M, Leaf P (1999). Past-year use of outpatient services for psychiatric problems in the National Comorbidity Survey. Am J Psychiatry.

[12] Maercker A, Forstmeier S, Wagner B, Glaesmer H, Brähler E (2008). Posttraumatische Belastungsstörungen in Deutschland: Ergebnisse einer gesamtdeutschen epidemiologischen Untersuchung. Nervenarzt.

[13] Perkonigg A, Kessler RC, Storz S (2000). Traumatic events and post-traumatic stress disorder in the community: prevalence, risk factors and comorbidity. Acta Psychiatr Scand.

[14] Tripp MM, Petrie TA (2001). Sexual abuse and eating disorders: A test of a conceptual model. Sex Roles.

[15] Weaver TL, Resnick HS, Kokoska MS, Louis S, Etzel JC (2007). Appearance-related residual injury, posttraumatic stress, and body image: Associations within a sample of female victims of intimate partner violence. J Traum Stress.

[16] Dyer AS, Borgmann E, Kleindienst N, Feldmann RE, Vocks S, Bohus M (2012). Body image in patients with posttraumatic stress disorder after childhood sexual abuse and co-occurring eating disorder. Psychopathology.

[17] Wittchen Z, Fydrich S (1997). Structured Clinical Interview for DSM-IV (SCID-I).

[18] Bernstein DP, Fink L, Handelsman L, Foote J, Lovejoy M, Wenzel K, Sapareto E, Ruggiero J (1994). Initial reliability and validity of a new retrospective measure of child abuse and neglect. Am J Psychiatry.

[19] Bernstein DP, Ahluvalia T, Pogge D, Handelsman L (1997). Validity of the Childhood Trauma Questionnaire in an adolescent psychiatric population. J Am Acad Child Adolesc Psychiatry.

[20] Vocks S, Legenbauer T, Wächter A, Wucherer M, Kosfelder J (2007). What happens in the course of body exposure? Emotional, cognitive, and physiological reactions to mirror confrontation in eating disorders. J Psychosom Res.

[21] Vocks S, Wächter A, Wucherer M, Kosfelder J (2008). Look at Yourself: Can body image therapy affect the cognitive and emotional response to seeing oneself in the mirror in eating disorders?. Eur Eat Disord Rev.

[22] Gast U, Rodewald F, Benecke HH, Driessen M (2001). Childhood Trauma Questionnaire – German version.

[23] Foa EB, Cashman L, Jaycox L, Perry K (1997). The validation of a self-report measure of posttraumatic stress disorder: The Posttraumatic Diagnostic Scale. Psychol Assessment.

[24] Griesel D, Wessa M, Flor H (2006). Psychometric qualities of the German version of the Posttraumatic Diagnostic Scale (PDS). Psychol Assessment.

[25] Stiglmayr C, Schmahl C, Bremner JD, Bohus M, Ebner-Priemer U (2009). Development and psychometric characteristics of the DSS-4-acute as a short instrument to assess dissociation during neuropsychological experiments. Psychopathology.

[26] Stiglmayr C, Braakmann D, Haaf B, Stieglitz R, Bohus M (2003). Entwicklung und psychometrische Charakterisierung der Dissoziations-Spannungs-Skala akut (DSS-akut). Psychother Psych Med.

[27] Cooper MJ, Fairburn CG (1992). Thoughts about eating, weight and shape in anorexia nervosa and bulimia nervosa. Behav Res Ther.

[28] Rüsch N, Schulz D, Valerius G, Steil R, Bohus M, Schmahl C (2011). Disgust and implicit self-concept in women with posttraumatic stress disorder and borderline personality disorder. Eur Arch Psy Clin N.

[29] Foa EB, Keane TM, Friedman MJ, Cohen JA (2009). Effective Treatments for PTSD: Practice Guidelines from the International Society for Traumatic Stress Studies.

[30] Resick P, Nishith P, Weaver T, Astin M, Feuer C (2002). A comparison of cognitive-processing therapy with prolonged exposure and a waiting condition for the treatment of chronic posttraumatic stress disorder in female rape victims. J Consult Clin Psych.

[31] Bohus M, Dyer AS, Priebe K, Krüger A, Kleindienst N, Schmahl C, Niedtfeld I, Steil R (2013). Dialectical behavior therapy for post-traumatic stress disorder after childhood sexual abuse in patients with and without borderline personality disorder: A randomised controlled trial. Psychother Psychosom.

